# A mixed methods systematic review exploring infant feeding experiences and support in women with severe mental illness

**DOI:** 10.1111/mcn.13538

**Published:** 2023-06-05

**Authors:** Natasha Baker, Debra Bick, Louise Bamber, Claire A. Wilson, Louise M. Howard, Ioannis Bakolis, Tayana Soukup, Yan‐Shing Chang

**Affiliations:** ^1^ Section of Women's Mental Health, Institute of Psychiatry, Psychology and Neuroscience, King's College London London UK; ^2^ Warwick Clinical Trials Unit, Warwick Medical School University of Warwick Coventry UK; ^3^ Child and Maternal Mental Health Team, South London and Maudsley NHS Foundation Trust London UK; ^4^ Department of Biostatistics and Health Informatics & Health Service and Population Research Department, School of Mental Health and Psychological Sciences, Institute of Psychiatry, Psychology and Neuroscience, King's College London London UK; ^5^ Department of Surgery and Cancer Imperial College London London UK; ^6^ Health Service and Population Research Department, Institute of Psychiatry, Psychology and Neuroscience, King's College London London UK; ^7^ Methodologies Research Division, Florence Nightingale Faculty of Nursing, Midwifery and Palliative Care, King's College London London UK

**Keywords:** breastfeeding, infant feeding, mixed methods, perinatal mental health, severe mental illness

## Abstract

There are many benefits of breastfeeding to women and their infants but meeting the recommended 6 months of exclusive breastfeeding is likely to be more challenging for women with severe mental illness (SMI). This is the first systematic review that aims to examine evidence of (a) infant feeding outcomes in women with SMI and the factors associated with this, (b) the experiences of infant feeding and infant feeding support for women with SMI, (c) interventions for supporting infant feeding among these women and (d) health care professionals' attitudes toward supporting infant feeding in women with SMI. Mixed methods systematic review was carried out using the principles of Joanna Briggs Institute's (JBI) 'convergent integrated' methodology. CINAHL, PsycINFO, Medline and MIDIRS were used to search literature between 1994 and 2022. The quality of selected articles was assessed using JBI critical appraisal tools and thematic synthesis was undertaken to obtain findings. Eighteen papers were included in the final review. Women with SMI were less likely to initiate and continue breastfeeding than women without SMI. Several challenges with breastfeeding were highlighted, and while these were often linked to women's mental health difficulties, inconsistent advice from health care professionals and poor support with breastfeeding further compounded these challenges. This review highlights that policy and practice need to take into account the individual challenges women with SMI face when planning, initiating and maintaining breastfeeding. Education and training for health care professionals are needed to enable them to provide tailored infant feeding support to women with SMI, which reflects their individual needs.

## Key messages

- Women with severe mental illness (SMI) are less likely to initiate and maintain breastfeeding compared to women without SMI.
- Challenges were identified among women with SMI including poor professional support with infant feeding.
- Education and training are required for health care professionals to individualise infant feeding support for women with SMI and should include information regarding medication and optimising sleep while breastfeeding.
- No evidence of effective interventions to support breastfeeding in women with SMI were identified and there was limited evidence of women's experiences of infant feeding.
- Further research is required with larger sample sizes and clearly defined measures of infant feeding outcomes.

## Graphical abstract

Women with severe mental illness (SMI) experience unique and complex challenges related to infant feeding and addressing these challenges requires an individualised approach within policy and practice. Future research is required with larger sample sizes, more robust measures of infant feeding, and interventions to support breastfeeding in women with SMI.

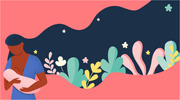

## 1 INTRODUCTION

Severe mental illness (SMI) is a collective definition for any mental health disorder which causes significant impairment to an individual's daily functioning and requires intensive input from mental health services. SMI in a perinatal context includes those with a pre‐existing illness who become pregnant, affecting around 2.8% of women, and those who develop a severe post‐partum psychiatric illness as their first episode, affecting 1–2 per 1000 births (Jones et al., 2014). The increased risks of SMI in the perinatal period are well acknowledged and suicide is the leading cause of direct maternal death in the first year post‐partum (Knight et al., 2022). SMI is also associated with increased risks of maternal morbidity and poor pregnancy outcomes including gestational diabetes (GDM), caesarean birth, placental abnormalities, preterm birth, low birth weight, growth restriction and admission to neonatal intensive care (Bennedsen et al., 2001; Frayne et al., 2019; Jablensky et al., 2005).

There are many benefits of breastfeeding for women and their infants and the evidence base in this area is well established. Breast milk reduces the risk of several childhood morbidities and neonatal infections linked to prematurity (Ip et al., 2007) and is particularly important for women with SMI, given their increased risk of poor pregnancy outcomes, including prematurity. There are several reasons why breastfeeding could be more challenging for this population, compared to those without SMI. The use of psychotropic medication and some symptoms of psychiatric illness can make women drowsy and unresponsive to infant feeding cues, while sleep deprivation associated with night‐time waking for feeds can be a trigger for mania and depression, exacerbating existing illnesses such as bipolar disorder (McIntyre et al., 2018). Despite this, being able to breastfeed remains important to many women with SMI, although their experiences of infant feeding and the support they receive with fulfilling breastfeeding intentions are unclear. There is some evidence to suggest that women with SMI feel a great sense of guilt if they cannot breastfeed due to prescribed medications (Dolman et al., 2016) and that perceived pressure to breastfeed can contribute to women abruptly stopping medication. For women with SMI, stopping psychotropic medication (with or without appropriate consultation with health care professionals [HCPs]) can make them more vulnerable to psychological deterioration (Lancet, 2012; Worsley et al., 2013).

Little is currently known about how women with SMI feed their babies or the factors associated with this. Within clinical practice, we know of no interventions to support infant feeding among women with SMI, or how HCPs support women with SMI. This systematic review therefore aimed to examine evidence of (a) infant feeding outcomes in women with SMI and the factors associated with this, (b) the experiences of infant feeding and infant feeding support for women with SMI, (c) interventions for supporting infant feeding among these women, and (d) HCPs attitudes toward supporting infant feeding in women with SMI. No previous reviews or protocols have been identified addressing these aims.

## 2 METHODS

A systematic review protocol was registered with PROSPERO: CRD42022353961. This mixed methods systematic review utilises the principles of Joanna Briggs Institute's (JBI) 'convergent integrated' methodology (Lizarondo et al., 2020). In this, quantitative findings are transformed into qualitative findings and converged into a single synthesis, allowing quantitative and qualitative data to be combined and interpreted together. The review aimed to answer the following questions:

Research questions:

1. How do women with SMI feed their infants in the first 2 years of life?
2. What factors are associated with infant feeding outcomes in infants of women with SMI?
3. How do women with SMI experience and view infant feeding practices and support?
4. Are there interventions to support breastfeeding in women with SMI and what is their effectiveness?
5. What are HCPs' attitudes toward supporting infant feeding in women with SMI?

### 2.1 Inclusion criteria

The PICOS (Population/Participants, Interventions/Phenomena of interest, Comparison/Context, Outcomes and Study types) framework adapted from JBI (Lizarondo et al., 2020) was used to develop the following inclusion criteria:

#### 2.1.1 Population

The study population included women aged 18 years and older, with SMI and a psychiatric episode occurring within 2 years after giving birth (psychiatric episode defined as: exacerbation of an existing illness or a new onset psychiatric episode, both requiring treatment by mental health services). For the purposes of this review, SMI was defined as either:

(A) Having a psychotic disorder (i.e. schizophrenia, schizoaffective disorder, bipolar affective disorder or depressive disorder with psychotic symptoms).
(B) Any other psychiatric condition defined as severe by a validated diagnostic instrument or from clinical records.
(C) Requiring input from secondary mental health services (psychiatry and specialist mental health services).

HCPs caring for women with SMI were also considered if the study reported their views and experiences of providing infant feeding support to women with SMI.

#### 2.1.2 Phenomena of interest

Studies were eligible for inclusion if they had reported infant milk feeding outcomes in infants under 2 years old, of women with SMI. This includes studies investigating the infant feeding intentions of women with SMI, breastfeeding initiation and duration, factors associated with breastfeeding outcomes and interventions to support breastfeeding in women with SMI. Qualitative studies were included if they reported the experiences and perceptions of infant feeding practices and support in women with SMI, or the views and experiences of HCPs supporting infant feeding in women with SMI. Studies investigating the safety of psychotropic medication while breastfeeding were excluded as were studies investigating infant feeding in patients with substance misuse disorder. Research investigating the use of donor milk and complementary feeding (infant receiving solid food in addition to breast milk at 6 months, also known as weaning) in the study population were also excluded as it was considered that these areas could hold very different meanings and experiences for women and would therefore require a separate study.

#### 2.1.3 Context

Only research conducted in high‐income countries (as defined by the World Bank classification) was included as poor mental health service infrastructure in low‐ and middle‐income countries can lead to difficulty in defining the severity of mental illness. Studies conducted in acute and primary care settings, including inpatient and community settings, or virtually and by telephone were included.

#### 2.1.4 Outcomes

The focus of this review included infant milk feeding outcomes in infants of women with SMI. This includes exclusive breastfeeding, defined by the World Health Organization (WHO & United Nations International Children's Emergency Fund [UNICEF], 2003) as an infant receiving only breast milk, and includes feeding exclusively expressed breast milk via a bottle. It also includes exclusive formula feeding and mixed feeding, which may involve giving some breastfeeds and some formula feeds. Outcomes also included infant feeding experiences of women with SMI, HCPs' attitudes towards infant feeding support of women with SMI and the effectiveness of interventions to support infant feeding in women with SMI.

#### 2.1.5 Types of studies

All experimental (e.g., randomised controlled trials and cluster randomised trials), quasi‐experimental studies and nonintervention, observational, qualitative and mixed methods studies were considered. Studies including primary and/or secondary data were included. Guidelines, policy papers, opinion pieces, commentary and discussion papers, reviews, conference proceedings and clinical case reports were excluded. Only studies published in English, since 1994 were included.

### 2.2 Search strategy

A search of Ovid MEDLINE(R) (1946); Maternity and Infant Care MIDIRS (1971); PsycINFO (1806); CINAHL (1982) was conducted using subject headings, MeSH terms and free‐text keywords. Boolean operators were used to combine terms. To ensure all relevant material was captured, a separate search of grey literature databases was undertaken in google scholar, Grey Literature Report, OpenGrey and WorldCat Dissertations and Theses using the phrase ‘infant feeding and mental health’. In addition, the reference lists of included papers were hand searched. Appendix A presents an example of the full search strategy for PsycINFO.

### 2.3 Study selection

Following the initial search of relevant databases, all citations were collated and imported into EndNote 20 (2021). All titles were screened for relevance by two independent reviewers (N. B. and Y. S. C.) and the abstracts of potentially eligible papers were then reviewed by N. B. Both reviewers independently screened the full texts of relevant papers to determine if they met the eligibility criteria. Any disagreement about eligibility was resolved by discussion and the final decision was agreed upon with a third reviewer (D. B.).

### 2.4 Assessment of methodological quality

The JBI critical appraisal instrument relevant to the study's design (Lockwood et al., 2020) was used to assess the quality of eligible studies. Each appraisal question reviewed in the paper was answered ‘yes’, ‘no’, ‘unclear’ or ‘not applicable’. All studies were included in data extraction and data synthesis, regardless of their methodological quality. Assessment of quality was independently appraised by N. B., Y. S. C. and L. B. and only Y. S. C. and L. B. assessed the Baker et al. (2021) paper to avoid conflict of interest. Any disagreements with the quality assessment were resolved via discussion or by DB if an agreement could not be met. ‘Supporting Information: Material A’ within the Supporting Information section details all answers to the JBI critical appraisal questions for each study design checklist. The assessment of the methodological quality was not used in the synthesis and interpretation of findings as the overall quality of papers was generally poor.

### 2.5 Data extraction

Quantitative and qualitative data extracted included study design, aim/objectives, sample size, study methods, population (including the psychiatric diagnosis under investigation where applicable), infant feeding measures used (where applicable) and key findings of significance to the review questions.

### 2.6 Data transformation, synthesis and integration

Following the principles of (JBI) ‘convergent integrated’ methodology (Lizarondo et al., 2020), quantitative data were converted into ‘qualitised’ data, meaning quantitative findings from included papers were interpreted and transformed into narrative descriptions to answer each review question. ‘Qualitised’ data and qualitative data were combined based on how they answered each individual review question, and then pooled together to produce themes. Thematic synthesis steps were based on those used by Chang et al. (2020) and are summarised as follows:

1. Data were extracted from findings of included studies
2. Extracted data were grouped for each review question and emergent themes identified
3. A list of themes was presented for each question
4. A synthesis of findings was produced

Variation in study designs, sample size and outcome measures meant meta‐analysis could not be performed.

## 3 FINDINGS

### 3.1 Study inclusion

**Figure 1:**
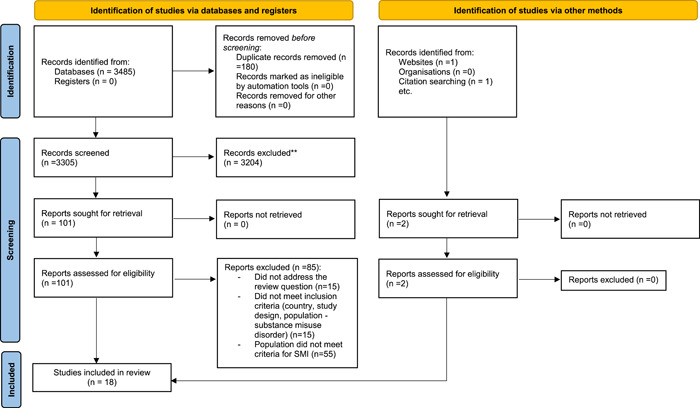
Preferred reporting items for systematic reviews and meta‐analyses flow diagram (2020).

An initial systematic search was carried out on 10 December 2021, and 3485 publications were identified (see Figure 1); one additional paper was identified from the grey literature search. Once duplicates were removed 3306 papers remained. Following title and abstract screening, 102 full texts were retrieved and reviewed independently by N. B. and Y. S. C. Eighty‐five papers were excluded at this stage for not meeting the inclusion criteria. The reference lists of eligible papers were searched and one further paper was identified. Searches were updated on 5 December 2022, but no further articles were identified.

### 3.2 Methodological quality

Though several studies were determined to lack methodological quality (Battle et al., 2006, 2008; Bergink et al., 2011; Hill et al., 2019; Lebedevs et al., 2020; Patel et al., 2005; Sakellari et al., 2020), we did not exclude these based on rigour. Two papers received a ‘yes’ to all critical appraisal questions (Artzi‐Medvedik et al., 2012; Challacombe et al., 2016), while the remaining studies had some methodological limitations (Baker et al., 2021; Bogen et al., 2010; Dolman et al., 2016; Martini et al., 2019; McCauley et al., 2014; Pearlstein et al., 2006; Taylor et al., 2022; Torgersen et al., 2010; Xu et al., 2014). Among the quantitative studies, this included unreliable outcome measures for infant feeding (Baker et al., 2021; McCauley et al., 2014), uncertainty if the outcome (infant feeding) was present at the start of the study (Baker et al., 2021; Martini et al., 2019; Torgersen et al., 2010; Xu et al., 2014) and unclear documentation of follow‐up (Baker et al., 2021; Torgersen et al., 2010; Xu et al., 2014). Qualitative studies did not define the theoretical perspectives of the research or the researcher's relationship to this (Dolman et al., 2016; Patel et al., 2005) and one qualitative study also lacked congruity generally across research methods (Patel et al., 2005). Please refer to ‘Supporting Information: Material A’ in the Supporting Information section for findings from the quality appraisal.

### 3.3 Characteristics of included studies

Eighteen papers were included in the review: 11 cohort studies, 2 cross‐sectional studies, 1 case series study, 1 case–control study, 2 qualitative studies and 1 quasi‐experimental study. The total sample size across all studies was *n* = 446,393, though this includes healthy controls and staff. The maximum sample size among the quantitative studies was *n* = 186,452 (Xu et al., 2014), and the minimum was *n* = 10 (McCauley et al., 2014). For qualitative studies, the maximum was *n* = 71 (Dolman et al., 2016), and the minimum was *n* = 21 (Patel et al., 2005). Two papers were from the same study (Battle et al., 2006, 2008). Most studies were based in the United Kingdom (five), the United States (four) and Australia (four), while the remaining four studies took place in Norway, Greece, Israel and the Netherlands. Most studies did not aim to investigate infant feeding in women with SMI but instead aimed to describe the characteristics, treatment and outcomes of women with SMI and their babies and reported infant feeding outcomes with a range of other outcome measures investigated (Battle et al., 2006; Bergink et al., 2011; Challacombe et al., 2016; Hill et al., 2019; Lebedevs et al., 2020; McCauley et al., 2014; Pearlstein et al., 2006). Similarly, one of the qualitative studies aimed to explore pregnancy decision‐making in women with bipolar disorder and reported incidental findings relating to women's experiences of infant feeding. The other qualitative study explored concerns about body shape in the post‐partum and reported findings relating to infant feeding (Patel et al., 2005). Two studies focused on HCPs' attitudes towards breastfeeding among women with schizophrenia (Artzi‐Medvedik et al., 2012; Sakellari et al., 2020). The remaining studies examined infant feeding intentions, outcomes and experiences of women with severe mental health disorders in the perinatal period including schizophrenia, severe depression, eating disorders (EDs), bipolar disorder and post‐partum psychosis (Baker et al., 2021; Battle et al., 2008; Bogen et al., 2010; Martini et al., 2019; Taylor et al., 2022; Torgersen et al., 2010; Xu et al., 2014). We found no studies which investigated interventions to support breastfeeding in women with SMI. Study characteristics (aim, design, methods, population, including diagnosis and key findings) are presented in Table 1.

**Table 1:** Study characteristics.

**Author, year, (country) Study design**	**Main aim(s)**	**Sample size and data collection methods**	**Population**	**Infant feeding measures**	**Data analysis methods**	**Key findings**
Artzi‐Medvedik et al. (2012) (Israel) A cross‐sectional study.	To survey attitudes among registered nurses regarding women with schizophrenia breastfeeding.	*N* = 110 Questionnaires	Midwives, mental health nurses and postnatal nurses working in Israel	A 49 item, self‐report questionnaire divided into clusters: sociodemographic characteristics; general professional characteristics; personal and professional experience, knowledge and attitudes regarding breastfeeding; personal and professional experience, knowledge, feelings and attitudes regarding schizophrenia; and personal and professional attitudes towards breastfeeding among women with schizophrenia (the primary outcome). The vignette was professional guidance for a woman with schizophrenia about breastfeeding	Pearson *χ* ^2^ test and *t*‐test. ANOVA Multivariate logistic regression	70% of the respondents held positive attitudes about breastfeeding in women with schizophrenia. ‘The highest positive responses were among midwives (80%) and the lowest among psychiatric nurses (62.2%).’ ‘66.3% of nurses with positive attitudes completed clinical specialist training compared with 42.4% of those with negative attitudes (*χ* ^2^ = 4.25, *p* = 0.039)’ ‘Among those with a positive attitude compared with those with a negative attitude, a higher proportion had an academic education (58.4% vs. 39.4%)’ Negative attitudes were more likely in respondents with wider knowledge of schizophrenia as a disorder or negative feelings towards women with schizophrenia
Baker et al. (2021) (UK) A mixed methods secondary data analysis of a cohort study	To examine antenatal infant feeding intentions and infant feeding outcomes of women requiring acute psychiatric care in the first post‐partum year.	*N* = 218 Structured interviews	Women >18 who required acute psychiatric care (psychiatric in‐patient ward, mother baby unit home treatment teams) during the first postnatal year: ICD‐10 psychiatric diagnosis used. SMI: Unipolar disorder, bipolar disorder schizophrenia, anxiety disorder, disorders associated with the puerperium (not elsewhere classified), personality disorder	Retrospective self‐report of infant feeding intentions and infant feeding outcomes during a structured research interview 1‐month following discharge from psychiatric services. Responses included either breast, mixed or formula feeding	Descriptive statistics, univariate analysis and multivariate regression analysis	'66.1% women reported breastfeeding in some capacity (42.2% breastfed exclusively, 23.8% mix fed) and 33.9% formula fed. 85.3% women had antenatal intentions to breastfeed and these women were significantly more likely to breastfeed than those who had not intended to breastfeed (OR 48.0, 95% CI: 11.1–211.0, *p* < 0.01). Non‐Caucasian ethnicity was significantly associated with breastfeeding, as was employment status. Free text analysis demonstrated inconsistent advice from health care providers about breastfeeding'
Xu et al. (2014) (Australia) A population based longitudinal cohort study	To investigate the relationship between early breastfeeding experience and clinically diagnosed post‐partum mental disorder and the interval between birth and first psychiatric hospital admission.	Total sample size *N* = 186,452 (*N* = 2940 (1.6%) *admitted to a psychiatric hospital in the 1st year*) Clinical records	All mothers who gave birth to a live infant between 2007 and 2008 in New South Wales, Australia; mothers with mental disorders were identified using the ICD‐10 diagnosis codes. SMI: unipolar disorder, bipolar disorder schizophrenia, anxiety disorder, acute psychotic episode, personality disorder, adjustment disorder, mental disorder due to substance misuse	Routinely collected midwifery data from electronic health records 'Infant feeding on hospital discharge'; options included 'breastfeeding', 'expressed breastmilk' and 'infant formula'	Survival analysis	'Compared with “full breastfeeding' mothers, mothers who did not breastfeed at the time of discharge from hospital or care following home birth were more likely to be admitted to the hospital in the first year post‐partum for schizophrenia (ARR = 2.0; 95% CI 1.3–3.1); bipolar affective disorders (ARR = 1.9; 95% CI 1.1–3.5) and mental illness due to substance use (ARR = 1.8; 95% CI 1.3‐2.5)'. 'However, non‐breastfeeding mothers were less likely to be admitted to the hospital with a diagnosis of anxiety disorders (ARR = 0.6; 95% CI 0.5–0.9) within 12 months after birth'. The mean time period before the first hospital admission with a diagnosis of any mental illness was 169 days for 'full breastfeeding' mothers, which was 32 days longer than the 137 days for non‐breastfeeding mothers (*p* < 0.05)'
Battle et al. (2006) (US) A cohort study	To describe the clinical, social and demographic characteristics of women who attend psychiatric day hospital during their pregnancy or first post‐partum year. To examine diagnostic comorbidity, levels of symptoms and functioning, prior psychiatric history And willingness to take psychotropic medication during their pregnancy or post‐partum months.	*N* = 500; *n* = 398 from a psychiatric mother baby day hospital; *n* = 102 from outpatient behavioural clinic Clinical records	Women >18 either pregnant or within the 1st year post‐partum receiving psychiatric treatment from 1 of 2 psychiatric health centres. SMI: MDD 80.6%, bipolar disorder 2.0%, dysthymic disorder 1.0%, panic disorder 4.6%, PTSD 1.8%, OCD 1.4%, generalised anxiety disorder—0.4% Adjustment disorder 3.4% Other Axis I disorder 4.8%	Breastfeeding binary yes/no from clinical records.	Descriptive statistics, Pearson *χ* ^2^ test and *t*‐tests	Breastfeeding Yes—25%, No—50%, missing 25% 'Among postpartum patients, a significantly smaller proportion of breastfeeding women (61%) took psychotropic medications, compared with post‐partum women who were not breastfeeding (86%)'.
Battle et al. (2008) (US) A cohort study	To identify characteristics of post‐partum breastfeeding women who take antidepressant medication.	*N* = 73 Clinical records	Subsample of previous study (Battle et al., 2006)—only participants who were post‐partum, breastfeeding and diagnosed with MDD.	Only breastfeeding women included (exposure)—breastfeeding from original study was a binary yes/no from clinical records.	Descriptive statistics Pearson *χ* ^2^ test and *t*‐tests	'Women who took antidepressants reported significantly more depressive symptoms and greater functional impairment, compared to patients who did not take medication. In addition, patients who took antidepressants were significantly more likely to have a diagnosis of severe (vs. mild/moderate) depression, more likely to report a psychiatric history during their intake interview, and more likely to have had previous experience with psychotropic medications'. 'Women who took antidepressants were significantly less likely to be in a committed relationship (*χ* ^2^: 4.7, *p* = <0.05). Although not statistically significant, women taking antidepressants were also more likely to be diagnosed with recurrent depression (*χ* ^2^: 3.7, *p* = 0.06). No associations were found in parity, age, ethnicity, and educational attainment'.
Bergink et al. (2011) (Netherlands) A prospective cohort study	To examine the risk factors, phenomenology, mode of onset and clinical course of PPP.	*N* = 51 patients (exposure—PPP) and *n* = 6969 controls (representing general population) Questionnaire	Control group from generation R study—gave birth between 2002 and 2006 in the same geographical location. Cases: females over 18 years old with a diagnosis of PPP admitted to the MBU in Rotterdam.	Clinician administered questionnaire including breastfeeding as a binary Yes/No.	Survival analysis	No differences found in prevalence of breastfeeding—controls: 87%, cases: 88.2% (OR 1.12, 95% CI 0.48–2.66).
Bogen et al. (2010) (US) A cohort study	To determine the relationships between (1) MDD and breastfeeding intention; (2) MDD and breastfeeding initiation and status at 2 and 12 weeks post‐partum; and (3) use of antidepressants and breastfeeding intention, initiation and breastfeeding status at 2 and 12 weeks post‐partum.	*N* = 168 Questionnaires	(1) Physically healthy women who were taking antidepressants during pregnancy for treatment of MDD, (2) pregnant women who had MDD at the time of enrolment but were not taking antidepressants and (3) women with no current major psychiatric disorder and no antidepressant treatment.	A feeding intention survey, created for the study was completed at each pregnancy visit (20, 30 and 36 weeks) Self‐report feeding practice surveys were completed at 2 and 12 weeks post‐partum. Clinicians also recorded women's infant feeding practices during study visits, including the proportion of feeds that were breast milk. The survey and clinician breastfeeding data were combined to determine breastfeeding status.	Pearson *χ* ^2^ and fishers exact test, ANOVA and logistic regression	Neither MDD nor depressive symptom severity in pregnancy was related to breastfeeding intention, initiation or duration at 2 and 12 weeks. Antidepressant use in pregnancy was negatively associated with breastfeeding intentions. Antidepressant use at 2 weeks post‐partum was negatively associated with 12‐week breastfeeding status.
Challacombe et al. (2016) (UK) A case–control study	To examine the impact of post‐partum OCD on parenting and mother‐infant interactions.	*N* = 74–37 cases (with OCD) and 37 controls (without OCD) Questionnaires and observations of interaction with infant	37 mothers with post‐partum OCD and their 6 month old infants were compared with 37 community control dyads. Women were diagnosed with severe OCD.	Self‐report	Regression analysis	27% of women with OCD were breastfeeding at 6 months, compared to 57% of controls (*p* = 0.01) Several women with OCD did not or felt they could not breastfeed because of the medication they were prescribed.
Hill et al. (2019) (Australia) A retrospective review of medical records (Cohort study)	To define the characteristics and treatments of women and their babies admitted to an MBU in Australia with PPP.	*N* = 25 Clinical records	Mothers with PPP admitted to an MBU.	Observation/medical records	Descriptive statistics	Breastfeeding on admission to MBU—56% Breastfeeding at discharge from MBU—36%
Lebedevs et al. (2020) (Australia) A review of medical records (Cohort study)	To define the management of perinatal women with a diagnosis of bipolar disorder, taking either lithium or sodium valproate and to investigate how this affected their plans to breastfeed.	*N* = 67 Clinical records	Women diagnosed with bipolar being treated with lithium and/or sodium valproate while admitted to an MBU in Australia between 2010 and 2018.	Observation/medical records	Descriptive statistics	Overall, 77.6% breastfed during admission to the MBU. 54 women (80.6%) expressed intention to breastfeed their infant before or immediately after delivery, but only 20 (29.9%) continued to breastfeed up to discharge from the MBU. 47.8% stopped breastfeeding during their MBU admission; 34.3% due to initiation of lithium therapy. Other reasons included breastfeeding being too stressful, poor milk supply, issue with latch at the breast, concern about other medications in breast milk, and weaning. A total of 15 (22.4%) patients did not breastfeed during their MBU admission. There were more women who breastfed while taking valproate than women taking lithium '(40.3% of women taking Valproate breastfed vs 1.5% of women taking lithium) (*p* < 0.05)'.
Martini et al. (2019) (UK) A cohort study	To investigate breastfeeding outcomes, infant feeding behaviours and attitudes to infant feeding in women with EDs compared to HCs.	*N* = 99 (C‐ED: *n* = 25, P‐ED: *n* = 28, HCls: 46) Questionnaires	Women with an active eating disorder (C‐ED) Women with a P‐ED and HCs with no history or C‐ED diagnoses using the Structured Clinical Interview for DSM IV‐TR axis I disorders (SCID‐I) P‐ED had a history of a DSM‐IV‐TR ED but did not meet criteria for and ED for at least 1 year before their current pregnancy.	Self‐reported breastfeeding at 8 weeks and 6 months postnatal Infant feeding questionnaire administered at 8 weeks and 6 months post‐partum to assess maternal feeding attitudes and practices.	Linear and logistic regression analysis	'60.6% of women were exclusively breastfeeding at 8 weeks (64.4% of HC, 66.7% of P‐ED and 52% of C‐ED), and 64% were exclusively or partially breastfeeding at 6 months (66.7% of HC, 73.9% of P‐ED, and 57.1% of C‐ED)'. 'Women with P‐ED reported less awareness about infant hunger and satiety cues compared with HC. Women with P‐ED also had increased odds of having high concern about their infant overeating or becoming overweight compared with HC'. 'Women with C‐ED had increased odds of having high concern about infant overeating or becoming overweight at 6 months only (OR = 8.1, 95% CI [2.2, 27.7]) compared with HC'.
McCauley et al. (2014) (Australia) A case series study	To investigate neonatal outcomes for 10 babies of women with SMI.	*N* = 10 Interviews	Babies of mothers with history of SMI—psychotic disorders (diagnosis unspecified), either pregnant or post‐partum who were taking or had recently taken antipsychotic medication.	The women participated in telephone or face‐to‐face interviews at 6 weekly intervals during pregnancy and after birth, at 6, 12 weeks, 6 and 12 months. And medical records	Case study methodology using tables, charts, figures and ratios	50% (5) breastfed, varying in duration and exclusivity—3 mothers breastfed for 12 months, 1 for 2 weeks and another mix fed.
Patel et al. (2005) (UK) Qualitative study	To compare how women with different levels of ED and women without ED perceive and cope with changes to their body shape and weight after having a baby.	*N* = 21 Questionnaire and Interviews	All women completed the ED examination—Questionnaire, women with high scores were approached to take part and had a diagnostic interview when their infant was 2 years. Following diagnostic interview, women fell into one of three groups; ED (*n* = 6) or ‘at risk for ED’ (*n* = 9) and a comparison group of HCs (*n* = 6)	Questionnaire included questions about how the mothers had fed their infant (breast, bottle or both) One of qualitative interview themes was the feeding relationship with their infant.	Thematic content analysis	'Although the mothers were not specifically asked about their feelings and attitudes in relation to the way they were feeding their infants, the topic of breastfeeding emerged spontaneously from the interviews'. Mothers with greater ED symptoms found the infants dependency on them uncomfortable, often wanting ‘space’. 'Mothers with significant eating problems also described their perceptions about breastfeeding mainly in relation to their own weight reduction and fears of not achieving this'. ‘I am annoyed that I have to carry on eating to provide milk for my baby. (at risk group)’ p. 357 ‘I dread giving up breastfeeding because of the weight gain. (Eating disorder group)’ p. 357
Pearlstein et al. (2006) (US) Quasi‐experimental	To evaluate the feasibility of a larger randomised controlled trial evaluating and comparing the clinical characteristics associated with IPT, sertraline only and a sertraline/IPT combination for women with post‐partum depression.	*N* = 23 Self‐selection of 1 of 3 treatment groups and questionnaires	‘Women with a diagnosis of MDD made by clinical interview were recruited from a psychiatric Day Hospital'. Women had moderate ‐ severe depressive symptoms at baseline, determined self‐report (EPDS, BDI) and clinician‐rated (HRSD) measures of depression.	Unclear	Fishers exact test, *t*‐tests and analysis of covariance	At the time of enrolment, approximately half of the study participants were breastfeeding (*n* = 12). There was a trend for breastfeeding women to opt for treatment without medication (66.7%) rather than treatment with medication (33.3%) (fishers 0.1).
Sakellari et al. (2020) (Greece) Cross‐sectional study	To explore health professionals’ (health visitors, midwives, nurses working in mental health care) attitudes towards breastfeeding among women with schizophrenia in Greece and to examine the validity and reliability of the Greek version of a specific rating scale on attitudes towards breastfeeding among women with schizophrenia	*N* = 170 Questionnaires	Participants were 40 midwives and 64 nurses who were working at psychiatric hospitals.	Scale developed by Artzi‐Medvedik et al. (2012)—'sociodemographic characteristics; general professional characteristics; personal and professional experience, knowledge and attitudes regarding breastfeeding; personal and professional experience, knowledge, feelings and attitudes regarding schizophrenia and the vignette patient; professional guidance for women with schizophrenia about breastfeeding; and personal and professional attitudes towards breastfeeding among women with schizophrenia'.	EFA PCA Pearson's *χ* ^2^ test, Fisher's exact test, *t*‐test and ANOVA	'Attitudes towards breastfeeding among women with schizophrenia were similar across the three groups'. 'Greater scores on attitudes towards women with schizophrenia and attitudes towards breastfeeding among women with schizophrenia were found in those that had previous contact with a person with schizophrenia. Furthermore, greater scores on attitudes towards women with schizophrenia were found in those that have provided consultation to a woman with schizophrenia on breastfeeding issues'.
Taylor et al. (2022) (Canada) Cohort study	To measure rates of maternal‐infant skin‐to‐skin contact and breastfeeding initiation at birth among mothers with schizophrenia, compared to women without schizophrenia, giving birth in Ontario, Canada.	Total population *n* = 218 906 mother–infant dyads: 471 (0.2%) with schizophrenia and 218 435 (99.8%) without. Clinical records	All live singleton deliveries occurring in‐hospital in Ontario from 1 April 2012, to 31 March 2014. Maternal schizophrenia status was established using a validated algorithm comprising: (1) one or more hospitalisations or (2) 3 or more outpatient physician visits within 3 years, for schizophrenia or schizoaffective disorder.	Hospital electronic health record data: Primary outcomes: (1) any mother–infant skin‐ to‐skin contact in the first 2 h after birth; (2) opportunity to initiate breastfeeding in first 2 h after delivery. Secondary outcomes: (3) intention to breastfeed; (4) in‐hospital assistance with breastfeeding within 6 h of delivery after breastfeeding initiation; (5) any breast milk from birth to discharge from hospital and (6) exclusive breast milk from birth to discharge from hospital.	Descriptive statistics, Modified poisson regression	Primary and secondary outcome (no schizophrenia vs. schizophrenia): (1) Skin to skin: 78.1% versus 65.2% (2) Breastfeeding initiation in first 2 h: 52.6% versus 38.9% (3) Intention to breastfeed: 92.6% versus 76.2% (4) Support with breastfeeding: 58.6% versus 44.7% (5) Any breastfeeding before discharge: 89.6% versus 74.4% (6) Exclusive breast milk at discharge: 63.3% versus 48.7%
Torgersen et al. (2010) (Norway) Cohort study	To explore the prevalence of breastfeeding across ED subtypes (AN, BN, BED and eating disorders not otherwise specified—purging subtype) compared to women with no ED during the first 6 months after birth.	*N* = 3,355 AN (39) BN (334) BED (2007) EDNOS‐P (42) Controls (NO ED) (36 933) Questionnaires	Pregnant women recruited to the Norwegian mother and child cohort study between 1999 and 2007. Women were either defined as having AN, BN, BED, EDNOS‐P or no ED.	At 6 months mothers self‐report breastfeeding, bottle feeding and supplementary feeding in the preceding 6 months.	One‐way ANOVA and survival analysis	98% of the mothers initiated breastfeeding overall, and there were no significant differences between the mothers with various EDs and mothers with no EDs. Breastfeeding initiation ED women: AN: 81% BN: 81% BED: 80% EDNOS‐P: 71% All ED groups had significantly elevated risk of breastfeeding cessation during the first 6 months post‐partum compared to women with no ED. After adjusting for confounders only mothers with AN (HR, 2.35; 95% CI, 1.22–4.53) and EDNOS‐P (HR, 1.95; 95% CI 1.08‐3.53) had increased risk for cessation of breastfeeding.
Dolman et al. (2016) (UK) Qualitative study	To explore factors influencing decision‐making in women with bipolar disorder regarding pregnancy and childbirth.	*N* = 21 (interviewees) *N* = 50 commented via online forum Interviews and comments via an online forum	Women with a diagnosis of bipolar disorder and either thinking about becoming pregnant, currently or recently pregnant.	Infant feeding was not formally asked about in the interviews but came up in some of the women's narratives spontaneously.	Thematic analysis	A subtheme of stigma included ‘stigma against women unable to breastfeed’. This stigma led to perceived pressure to breastfeed and influenced decisions the women made about treatment such as not taking medication postnatally due to breastfeeding. ‘I got a lot of “Why aren't you breastfeeding? Why?” … I never heard them talk to anyone else like that’ Stigma about not breastfeeding due to their bipolar disorder was also perceived to lead to poor help and support with formula feeding. 'Nobody showed us how to feed her [with a bottle] and we hadn't a clue'

Abbreviations: AN, anorexia nervosa; ANOVA, analysis of variance; ARR, adjusted relative risk; BDI, Beck's depression inventory; BED, binge eating disorder; BN, bulimia nervosa; CI, confidence interval; C‐ED, current ED; ED, eating disorder; EDNOS‐P, ED not otherwise specified—purging subtype; EFA, exploratory factor analysis; EPDS, Edinburgh postnatal depression scale; HC, healthy control; HR, hazard ratio; HRSD, Hamilton rating scale for depression; ICD, International classification of diseases; IPT, interpersonal psychotherapy; MBU, Mother and Baby Unit; MDD, major depressive disorder; OCD, obsessive‐compulsive disorder; OR, odds ratio; PCA, principal component analysis; P‐ED, past ED; PPP, post‐partum psychosis; PTSD: post‐traumatic stress disorder; SMI, severe mental illness.

### 3.4 How do women with SMI feed their infants in the first 2 years of life?

Three papers reported infant feeding intentions compared to actual infant feeding outcomes, and five papers reported the duration of breastfeeding. A common limitation was that very few studies specified if breastfeeding in the sample was exclusive or nonexclusive (mixed feeding). Therefore, breastfeeding prevalence in this section denotes ‘any’ breastfeeding, which is breastfeeding irrespective of exclusivity. Two themes were generated from 11 studies and the following section provides an overview of the two themes: breastfeeding prevalence and duration among women with SMI.

#### 3.4.1 Breastfeeding prevalence among women with SMI

Eleven studies reported breastfeeding outcomes but only four papers defined if this had been exclusive or nonexclusive breastfeeding (Baker et al., 2021; Martini et al., 2019; McCauley et al., 2014; Taylor et al., 2022). Breastfeeding prevalence was not the primary outcome of investigation in 7 of the 11 studies (Battle et al., 2006; Bergink et al., 2011; Challacombe et al., 2016; Hill et al., 2019; Lebedevs et al., 2020; McCauley et al., 2014; Pearlstein et al., 2006). Most aimed to investigate the characteristics and management of women with SMI in the perinatal period and reported infant feeding outcomes together with other outcomes. The ‘any’ breastfeeding prevalence within the literature reviewed varied significantly from as low as 25% in Battle et al. (2006)'s cohort study of 500 women with SMI, attending psychiatric outpatient clinics in the United States, to as high as 88% in Bergink et al. (2011)'s cohort study of 51 women with post‐partum psychosis, admitted to a psychiatric inpatient facility in the Netherlands (see Table 1 for study breastfeeding rates). Breastfeeding prevalence among the studies in this review varied too much to indicate any differences between different psychiatric diagnoses. However, two studies which compared infant feeding outcomes to those of a control group found that breastfeeding was lower among women with SMI than in women without SMI (Challacombe et al., 2016; Taylor et al., 2022).

#### 3.4.2 Breastfeeding duration among women with SMI

The majority of studies did not specify when the infant feeding method had been recorded and only four studies discussed how long participants had breastfed. Hill et al. (2019) and Lebedevs et al. (2020) explored breastfeeding cessation during Mother and Baby Unit (MBU) admission in Australia. Hill focused specifically on women with post‐partum psychosis, while Lebedevs focused on women with bipolar disorder. Hill reported that a large proportion of women stopped breastfeeding during MBU admission (56% breastfeeding at admission and 36% at discharge) but did not investigate the reasons for this. Lebedevs also reported that only 29.9% were still breastfeeding at discharge from the MBU (a total of 77.6% breastfed during admission) and that most women in the study ceased breastfeeding due to starting lithium therapy. Women also stopped breastfeeding because of concerns about other psychotropic medications they were prescribed, if they found breastfeeding too stressful or were having breastfeeding difficulties such as insufficient milk supply or poor latch.

Martini et al. (2019) and Torgersen et al. (2010) investigated breastfeeding outcomes in women with EDs, compared to women with no history of ED. In both studies, no differences were found in breastfeeding initiation among cases and controls. Martini also found little difference in breastfeeding outcomes at 6 months post‐partum, the frequency of which remained around 60%. However, in Torgerson's study women with EDs had a significantly higher risk of stopping breastfeeding before 6 months post‐partum than women without EDs.

### 3.5 What factors are associated with infant feeding outcomes in infants of women with SMI?

Seven studies described characteristics associated with breastfeeding and difficulties relating to mental illness which make breastfeeding more challenging. Three themes were generated from the seven studies and are discussed under the following subheadings.

#### 3.5.1 Intentions to breastfeed

Four studies reported the breastfeeding intentions of women in their sample. Psychiatric diagnosis varied among these studies and included schizophrenia, bipolar disorder and major depressive disorder (MDD) (details can be found in Table 1). Baker et al. (2021) and Lebedevs et al. (2020) found that most women had planned to breastfeed (85.3% and 80.6%, respectively) and intending to breastfeed was identified as having a positive influence on the likelihood of actually breastfeeding and on breastfeeding exclusively (Baker et al., 2021 & Bogen et al., 2010). Bogen et al. (2010) also found that intending to breastfeed exclusively and being very determined to breastfeed were significant predictors of breastfeeding outcomes in women with MDD. However, Taylor et al. (2022) found that women with schizophrenia were less likely to intend to breastfeed than women without schizophrenia and were also less likely to initiate breastfeeding.

#### 3.5.2 Mental health treatment

Mental health treatment, particularly with psychotropic medication, was identified in four studies as a barrier to breastfeeding and decisions about infant feeding were subsequently more difficult for women (Baker et al., 2021; Battle et al., 2006; Challacombe et al., 2016; Lebedevs et al., 2020). However, a bidirectional relationship was observed between infant feeding choices and treatment with medication. While, treatment with medication can influence women's choices around infant feeding, breastfeeding can also impact mental health treatment choices. Both Battle et al. (2006) and Pearlstein et al. (2006) found that breastfeeding women with MDD often preferred treatment without medication, potentially impacting their own mental wellbeing and psychological recovery.

#### 3.5.3 Maternal background characteristics

Women's background characteristics, including sociodemographic, clinical and lifestyle characteristics were found to be associated with infant feeding outcomes in two studies. Baker et al. (2021) found that women from Black, Asian and other minority ethnic (BAME) backgrounds in the United Kingdom were more likely to report breastfeeding compared to White women (adjusted odds ratio [AOR] 3.1, 95% confidence interval [CI]: 1.2–8.1) and women who were employed before their pregnancy were more likely to breastfeed (AOR 2.5, 95% CI:1.1–5.8). Similarly, Bogen et al. (2010) found evidence of an association between ethnicity and breastfeeding, though, in their cohort study in the United States, women identifying as White were more likely to breastfeed than Black and Latina women (*p* = 0.009). Additionally, Bogen et al. (2010) found that higher levels of maternal education, prior breastfeeding experience and nonsmoking status were positively associated with breastfeeding. While plans to return to work earlier and obesity were negatively associated with breastfeeding initiation.

### 3.6 How do women with SMI experience and view infant feeding practices and support?

Six studies reported the infant feeding experiences of women with SMI which were often complicated by their mental health condition, particularly if requiring medication. Three of these studies also described women's experiences of infant feeding support which were often negative. Three themes were generated from six studies and are discussed under the following subheadings.

#### 3.6.1 Poor infant feeding support and advice from health care professionals

In two of the studies, Inaccurate, contradictory or insufficient advice from HCPs about breastfeeding while taking psychotropic medication was described as a key issue for women (Baker et al., 2021; Dolman et al., 2016). This made infant feeding choices more challenging and resulted in discontinuing breastfeeding earlier than planned. Baker et al. (2021)'s mixed methods secondary data analysis presented some of the findings from free‐text comments and several women in the study described contradictory advice from HCPs. For example:

Was on Quetiapine in pregnancy. Psychiatrist said I could not breastfeed on the medication but OB/GYN said I could (Baker et al., 2021, p. 6).

In Dolman et al. (2016)'s qualitative study, women perceived that stigma surrounding their bipolar disorder and the need for medication prevented them from receiving support with formula feeding:

Nobody showed us how to feed her [with a bottle] and we hadn't a clue (Dolman et al., 2016) (p. 297)

Taylor et al. (2022) also found that women with schizophrenia received less support with breastfeeding than women without schizophrenia. However, it was not made clear how support differed for women with schizophrenia and support was only described as ‘in‐hospital assistance with breastfeeding within 6 hrs of delivery, after breastfeeding initiation’ (p. 147).

Women who were receiving in‐patient psychiatric care considered that psychiatric treatment was not conducive to breastfeeding, which was commonly attributed to the need for medication and the psychiatric inpatient environment. Even when women reported positive experiences of inpatient care, breastfeeding support was still described as negative: 'The nursery staff were excellent and taught me so much. The only lack of support came from my desire to start breastfeeding again, I didn't get any support or encouragement' (Baker et al., 2021, p. 8).

#### 3.6.2 Difficult infant feeding choices and experiences while taking psychotropic medication

An important finding observed across the data is the impact psychotropic medication can have on women's decision‐making around infant feeding, which was highlighted in seven studies. Stopping breastfeeding earlier than planned due to misinformation or requiring medication contraindicated during lactation was distressing for women and often had a negative impact on their mental health: 'I had to go on olanzapine which meant I had to stop breastfeeding, this had a massive negative impact' (Baker et al., 2021, p. 6). Both Baker et al. (2021) and Dolman et al. (2016) found this led to women disregarding medical advice and stopping medication to breastfeed which can have very negative implications for women with SMI, such as relapse. Battle et al. (2006) also found that women with MDD often declined treatment with medication to breastfeed, although this was dependent on the severity of their symptoms, their previous experience with psychotropic medications and whether they were in a stable relationship.

#### 3.6.3 The distinct features of infant feeding for women with EDs

Two studies investigated attitudes towards infant feeding in women with EDs. Both Martini et al. (2019) and Patel et al. (2005) found that motivations for breastfeeding and subsequent infant feeding behaviours are unique to this particular psychopathology. The infant feeding outcomes and beliefs of women with EDs in both studies were compared against healthy controls with no history of ED and despite no significant differences in breastfeeding rates among women with and without EDs, differences in women's perceptions of breastfeeding were identified. Women with current and past EDs were less aware of their baby's feeding cues and were more concerned about their infants' overeating than women without EDs (Martini et al., 2019). In Patel et al. (2005)'s qualitative study, women with EDs felt unhappy about their infants' continued dependency on them for nutrition 'I am annoyed that I have to carry on eating to provide milk for my baby' (Patel et al., 2005, p. 357). Though others were motivated to breastfeed as they believed this may help them to lose weight postnatally: 'I dread giving up breastfeeding because of the weight gain' (Patel et al., 2005, p. 357).

### 3.7 What are HCPs’ attitudes towards supporting infant feeding in women with SMI?

Two studies conducted in Israel and Greece focused on HCPs’ attitudes towards breastfeeding among women with schizophrenia (Artzi‐Medvedik et al., 2012; Sakellari et al., 2020). Staff included mental health nurses, midwives and health visitors. Two themes were generated from these two studies and are discussed under the following:

#### 3.7.1 Overall positive attitudes from HCPs towards breastfeeding among women with schizophrenia

The majority of HCPs held positive views about breastfeeding among women with schizophrenia, with some variation between different staff groups. Artzi‐Medvedik et al. (2012) found that midwives in Israel held the most positive views, while psychiatric nurses had the least positive views. In Sakellari et al. (2020)'s study from Greece, all staff groups held similar positive attitudes about breastfeeding among women with schizophrenia but psychiatric nurses had more positive views when compared to health visitors.

#### 3.7.2 Factors affecting views on breastfeeding among women with schizophrenia

Variation in attitudes were found to be influenced by HCPs’ own professional experience, their education and training and their own views about people with schizophrenia. Artzi‐Medvedik et al. (2012) found that nurses with greater academic education, including a master's degree had more positive attitudes to breastfeeding among women with schizophrenia, while nurses with more knowledge and experience of schizophrenia as a disorder were less likely to have a positive attitude. Conversely, Sakellari et al. (2020) found that attitudes were more positive in staff who had previous contact with a person with schizophrenia and among staff who had experience of advising women with schizophrenia about breastfeeding.

## 4 DISCUSSION

This review explored qualitative and quantitative evidence about infant feeding practices and infant feeding support experienced by women with SMI. Findings have highlighted that infant feeding choices for women with SMI are often complex, with barriers preventing women from initiating and maintaining breastfeeding including; the use of psychotropic medication, poor or inconsistent advice and support from HCPs and concerns around resuming restrictive eating in women with EDs. These findings are discussed within the context of the wider literature and implications for policy, practice and future research considered.

### 4.1 Infant feeding outcomes

The prevalence of breastfeeding varied across the studies, but in general, breastfeeding rates were lower in women with SMI than in women without SMI. The data presented in this review cannot be used to accurately define the prevalence of breastfeeding among women with SMI due to the heterogeneity of outcome measures used and the methodological limitations of included studies. Globally, 65% of infants are exclusively breastfed for the first 2 days after birth (UNICEF, 2021) and in the United Kingdom, the prevalence of breastfeeding initiation is 74.2% (Royal College of Obstetricians and Gynaecologists, 2023). While both are higher than rates reported in most of the studies in this review, it was unclear from included papers if they were reporting breastfeeding initiation or breastfeeding at another time point.

To date, much of the literature in this field has focused on women with common mental health disorders (CMHDs), although findings were similar to those in this review. For example, CMHDs like depression and anxiety are also associated with reduced breastfeeding intention and initiation rates, nonexclusivity and shorter duration of breastfeeding (Amiel Castro et al., 2017; Deave et al., 2008; Fallon et al., 2016; Galbally et al., 2019). Similarly to our findings, early cessation of breastfeeding has also been demonstrated in women with posttraumatic stress disorder (Garthus‐Niegel et al., 2018) and in women admitted to MBUs with psychotic disorders in India (Chandra et al., 2015). Interestingly, 86% of Chandra's cohort resumed breastfeeding following psychiatric recovery which was not demonstrated in any of the literature in our review. Adherence to WHO recommendations for 6 months of exclusive breastfeeding is low in the general population (McAndrew et al., 2012). However, our findings highlight that in women with SMI, a shorter duration of breastfeeding could be associated with the competing needs of mental health treatment and poor support from HCPs. This emphasises the need for individualised infant feeding support in this population.

### 4.2 Factors associated with infant feeding choices and outcomes

While breastfeeding rates appear to be lower in women with SMI than in women without SMI, the predictors of breastfeeding are similar to those demonstrated in the wider literature. Breastfeeding initiation and duration are predicted by demographic factors such as maternal age, ethnicity and socioeconomic status (McAndrew et al., 2012) and by clinical characteristics (body mass index, smoking status), social and professional support (Amiel Castro et al., 2017; Chang et al., 2020; Scott et al., 2006). We found similar findings were reported by Baker et al. (2021) and Bogen et al. (2010) though outcomes relating to ethnicity and breastfeeding differed between the two studies. This is perhaps more reflective of racial disparities in infant feeding trends generally in the United States, compared to the United Kingdom. For example, in the United States, unlike the United Kingdom, more White women breastfeed compared with Black, Asian and Latina women (Jones et al., 2015).

The strongest predictor of breastfeeding reported by Baker and by Bogen was maternal breastfeeding intentions. Likewise, intending to breastfeed is strongly associated with breastfeeding outcomes in women without mental health disorders reported in the wider literature (Amiel Castro et al., 2017; Scott et al., 2004, 2006). However, unmet breastfeeding intentions and breastfeeding difficulties have also been found to increase depressive symptoms (Borra et al., 2015; Chang et al., 2023; Chaput et al., 2016; Gregory et al., 2015; Rivi et al., 2020). Although we did not find the same link, there was evidence that breastfeeding could be protective against a severe psychiatric episode requiring hospital admission (Xu et al., 2014). Despite this, we found that women experienced multiple challenges with breastfeeding and did not feel supported (Baker et al., 2021; Dolman et al., 2016; Taylor et al., 2022). These findings emphasise the importance of providing dedicated infant feeding support to women with SMI and highlight that HCPs need to provide compassionate care to women with unfulfilled breastfeeding intentions and support them with alternative options to breastfeeding.

### 4.3 Women's experiences of infant feeding

Few qualitative studies have explored the infant feeding experiences of women with SMI. Baker et al. (2021) revealed the struggle women face in making infant feeding choices, particularly when taking psychotropic medication. Martini et al. (2019) and Patel et al. (2005) provided insights into the feeding motivations and perceptions of women with EDs, which were often strongly linked with behaviours and beliefs underpinning their ED. Stapleton et al. (2008) also investigated the infant feeding decisions of women with EDs and demonstrated similar findings, though this study did not meet the inclusion criteria of our review.

Our review found that difficulties with infant feeding were compounded by inconsistent advice from HCPs and poor support with breastfeeding. In the wider literature, women experiencing postnatal depression and other emotional difficulties in the post‐partum period have also highlighted that poor support with breastfeeding and pressure to breastfeed enhanced their distress (Coates et al., 2014; Da Silva Tanganhito et al., 2020). Likewise, feelings of guilt and inadequacy as a mother due to not breastfeeding have previously been reported (Fallon et al., 2017; Humphries & McDonald, 2012). Dolman et al. (2016)'s study, included in our review highlights that this can add additional stigma for women with mental illness. Women with mental illness find the ‘breast is best’ rhetoric unhelpful and want more problem‐solving support with breastfeeding rather than the promotion of the benefits (Coates et al., 2014). Fallon et al. (2017) reflect that emotions linked to infant feeding can be a precursor to more serious post‐partum mood disorders and highlights the potential emotional cost of trying to achieve the current WHO guidance. It has been suggested that the current recommendations do not account for individual circumstances such as SMI (Lagan et al., 2014) and emphasises Humphries’ argument that ‘it is important to explore what extent women struggling with mental health issues are able to successfully navigate the territory of best practice in the post‐partum period’ (Humphries & McDonald, 2012, p. 382).

### 4.4 HCPs’ attitudes and confidence with infant feeding support

Findings relating to poor support and inconsistent breastfeeding advice could reflect a lack of awareness and limited confidence in feeding support for women with SMI experienced by HCPs. In the wider literature, Kean et al. (2013) found that out of 68 psychiatrists surveyed about prescribing practices for pregnant and lactating women, the majority (*n* = 42, 61.7%) felt they needed more training around prescribing medication to breastfeeding women. Mental health nurses also report limited confidence to support infant feeding, having little experience and feeling uneasy due to limited evidence and fear of potential legal and ethical implications (McConachie & Whitford, 2009). Likewise, midwives also lack confidence in this area and want additional training to support women with SMI (Noonan et al., 2018). In the current review, both Artzi‐Medvedik et al. (2012) and Sakellari et al. (2020) explored HCPs’ attitudes towards breastfeeding among women with schizophrenia. The findings from both studies emphasise that HCPs involved in the perinatal care of women with SMI require enhanced infant feeding training to better support women. This should include information regarding psychotropic medication use and optimising sleep while breastfeeding, to prevent relapse.

In summary, although several studies in this review reported infant feeding outcomes, the prevalence of breastfeeding, including breastfeeding exclusivity and duration among women with SMI remains poorly understood. Qualitative evidence was limited and no research was identified which addressed interventions to support breastfeeding. Intervention studies, investigating ways to support infant feeding among women with SMI should be designed to address this particular evidence gap. The weakness of the evidence base also indicates that further research in this area is required with larger sample sizes, longitudinal cohorts with large follow up and clearer measures of infant feeding outcomes. Although the current review excluded research investigating the use of donor milk and complementary feeding in women with SMI, these are also important areas for future research. An urgent review of current policy and practice is required to address and take into account the individual challenges women with SMI face when making decisions about feeding their infants.

### 4.5 Strengths and limitations

To our knowledge, this is the first systematic review to address the infant feeding practices and experiences of women with SMI. This review provides novel synthesised findings and specific insight into the experiences of women with SMI. A highlight of the review is that it includes quantitative and qualitative perspectives, offering a wider and more diverse understanding of this phenomenon. However, infant feeding was not the primary outcome of investigation in several studies and findings relating to feeding were minimal. Several studies had methodological limitations including small sample sizes and infant feeding measures were generally not robust. Breastfeeding exclusivity was also unclear, as was the time point infant feeding had been recorded postnatally, and caution must therefore be applied to the findings. We excluded non‐English language studies, studies published before 1994 and studies carried out in low‐ and middle‐income countries which may have introduced selection bias. Nevertheless, this review explores the literature base around infant feeding in the context of SMI which thus far has been widely underresearched. This review provides further understanding for researchers and clinicians in this field and indicates some possible areas for future research and improvements to care.

## 5 CONCLUSION

Women with SMI are less likely to initiate and maintain breastfeeding, compared to women without mental health disorders and they experience multiple breastfeeding barriers. Although barriers were commonly associated with women's specific mental health difficulties, poor infant feeding support and inconsistent information about breastfeeding was a universal finding. Addressing challenges experienced by women, including poor support, requires urgent consideration within policy and practice.

## AUTHOR CONTRIBUTIONS

All authors were involved in the conceptualisation and design of the review. Natasha Baker, Debra Bick and Yan‐Shing Chang conducted the search and assessed the selected papers for eligibility. Natasha Baker, Debra Bick, Louise Bamber and Yan‐Shing Chang performed the quality appraisal and extracted data. Natasha Baker drafted the manuscript and all authors provided feedback. All authors read and approved the final version of the manuscript.

## CONFLICT OF INTEREST STATEMENT

The authors declare that one of the papers included and critiqued in this review was first authored by N. B. (Baker et al., 2021).

## Supporting information

Supporting information.

Supporting information.

## Data Availability

The data that support the findings of this study are available from the corresponding author upon reasonable request.
