# Supplementary material for: A mixed methods systematic review exploring infant feeding experiences and support in women with severe mental illness
Source: Matern Child Nutr. 2023 Jun 5;19(4):e13538. doi: 10.1111/mcn.13538 (PMC10483956; doi:10.1111/mcn.13538)
Supplement: Supplementary file 1 — Supporting information. [file MCN-19-e13538-s001.docx]

**Appendix A:** Full search strategy for PsycINFO (1806)

| 1. | exp Breast Feeding/ |
| --- | --- |
| 2. | breast fe*.mp. |
| 3. | infant fe*.mp. |
| 4. | exp Bottle Feeding/ |
| 5. | formula fe*.mp. |
| 6. | cup fe*.mp. |
| 7. | expressed breast.mp. |
| 8. | exp Lactation/ |
| 9. | exp Perinatal Period/ |
| 10. | exp Postnatal Period/ |
| 11. | matern*.mp. |
| 12. | mother*.mp. |
| 13. | (severe mental illness or SMI).mp. |
| 14. | (severe mental disorder or serious mental disorder).mp. |
| 15. | exp Affective Disorders/ |
| 16. | severe depres*.mp. |
| 17. | puerperal psychosis.mp. or exp Postpartum Psychosis/ |
| 18. | bipolar.mp. or exp Bipolar Disorder/ or exp Bipolar II Disorder/ or exp Bipolar I Disorder/ |
| 19. | exp Eating Disorders/ or ED.mp. |
| 20. | exp Anorexia Nervosa/ or exp Bulimia/ |
| 21. | PTSD.mp. or exp Posttraumatic Stress Disorder/ |
| 22. | exp Obsessive Compulsive Disorder/ or OCD.mp. or exp Anxiety Disorders/ |
| 23. | exp Personality Disorders/ |
| 24. | exp Major Depression/ |
| 25. | postnatal*.mp. |
| 26. | postpartum.mp. |
| 27. | 1 or 2 or 3 or 4 or 5 or 6 or 7 or 8 |
| 28. | 9 or 10 or 11 or 12 or 25 or 26 |
| 29. | exp Mental Disorders/ |
| 30. | exp Schizophrenia/ |
| 31. | psychotic disorder.mp. |
| 32. | 13 or 14 or 15 or 16 or 17 or 18 or 19 or 20 or 21 or 22 or 23 or 24 or 29 or 30 or 31 |
| 33. | (mania or manic).mp. |
| 34. | 32 or 33 |
| 35. | self harm*.mp. |
| 36. | suicid*.mp. |
| 37. | 34 or 35 or 36 |
| 38. | (maternal mental health or maternal mental illness or perinatal mental health). |
| 39. | 37 or 38 |
| 40. | 27 and 28 and 39 |
| 41. | limit 40 to (english language and yr="1994 - 2021") |
