# Supplementary material for: A mixed methods systematic review exploring infant feeding experiences and support in women with severe mental illness
Source: Matern Child Nutr. 2023 Jun 5;19(4):e13538. doi: 10.1111/mcn.13538 (PMC10483956; doi:10.1111/mcn.13538)
Supplement: Supplementary file 2 — Supporting information. [file MCN-19-e13538-s002.docx]

| **Cohort studies** | **1. Were the two groups similar and recruited from the same population?** | **2. Were the exposures measured similarly to assign people to both exposed and unexposed groups?** | **3. Was the exposure measured in a valid and reliable way?** | **4. Were confounding factors identified?** | **5. Were strategies to deal with confounding factors stated?** | **6. Were the groups/participants free of the outcome at the start of the study (or at the moment of exposure)?** | **7. Were the outcomes measured in a valid and reliable way?** | **8. Was the follow up time reported and sufficient to be long enough for outcomes to occur?** | **9. Was follow up complete, and if not, were the reasons to loss to follow up described and explored?** | **10. Were strategies to address incomplete follow up utilized?** | **11. Was appropriate statistical analysis used?** |
| --- | --- | --- | --- | --- | --- | --- | --- | --- | --- | --- | --- |
| **Baker et al. (2021)** | Yes | N/A | Yes | Yes | Yes | Unclear | No | Yes | Unclear | Yes | Yes |
| **Xu et al. (2014)** | No | Yes | Yes | Yes | Yes | Unclear | Yes | Yes | Unclear | Yes | Yes |
| **Battle et al. (2006)** | No | Yes | Yes | No | No | Unclear | Unclear | Unclear | Unclear | Unclear | Yes |
| **Battle et al. (2008)** | N//A | N/A | Yes | No | No | Unclear | Yes | Unclear | Unclear | Unclear | Unclear |
| **Bergink et al. (2011)** | No | No | Yes | Yes | Yes | Unclear | Unclear | Unclear | Unclear | Unclear | Yes |
| **Bogen et al. (2010)** | Unclear | Yes | Yes | Yes | Yes | Yes | Yes | Yes | Yes | Yes | Yes |
| **Hill et al. (2019)** | N/A | N/A | Yes | Unclear | No | Unclear | Unclear | Unclear | Unclear | Unclear | No |
| **Lebedevs et al. (2020)** | N/A | Yes | Yes | No | No | Unclear | Unclear | Unclear | Unclear | Unclear | No |
| **Martini et al. (2019)** | No | Yes | Yes | Yes | Yes | Unclear | Yes | Yes | Yes | Yes | Yes |
| **Torgersen et al. (2010)** | No | Yes | Yes | Yes | Yes | Unclear | No | Yes | Unclear | Unclear | Yes |
| **Taylor et al. (2022)** | No | Yes | Yes | Yes | Yes | Yes | Yes | Unclear | Yes | Yes | Yes |

| **Cross-sectional studies** | **1. Were the criteria for inclusion in the sample clearly defined?** | **2. Were the study subjects and the setting described in detail?** | **3. Was the exposure measured in a valid and reliable way?** | **4. Were objective, standard criteria used for measurement of the condition?** | **5. Were confounding factors identified?** | **6. Were strategies to deal with confounding factors stated?** | **7. Were the outcomes measured in a valid and reliable way?** | **8. Was appropriate statistical analysis used?** |  |  |  |
| --- | --- | --- | --- | --- | --- | --- | --- | --- | --- | --- | --- |
| **Artzi-Medvedik et al. (2012)** | Yes | Yes | Yes | Yes | Yes | Yes | Yes | Yes |  |  |  |
| **Sakellari et al. (2020)** | Unclear | No | Unclear | Unclear | Unclear | Unclear | Yes | Yes |  |  |  |
| **Case control studies** | **1. Were the groups comparable other than the presence of disease in cases or the absence of disease in controls?** | **2. Were cases and controls matched appropriately?** | **3. Were the same criteria used for identification of cases and controls?** | **4. Was exposure measured in a standard, valid and reliable way?** | **5. Was exposure measured in the same way for cases and controls?** | **6. Were confounding factors identified?** | **7. Were strategies to deal with confounding factors stated?** | **8. Were outcomes assessed in a standard, valid and reliable way for cases and controls?** | **9. Was the exposure period of interest long enough to be meaningful?** | **10. Was appropriate statistical analysis used?** |  |
| **Challacombe et al. (2016)** | Yes | Yes | Yes | Yes | Yes | Yes | Yes | Yes | Yes | Yes |  |

| **Quasi-experimental studies** | **1. Is it clear in the study what is the ‘cause’ and what is the ‘effect’ (i.e. there is no confusion about which variable comes first)?** | **2. Were the participants included in any comparisons similar?** | **3. Were the participants included in any comparisons receiving similar treatment/care, other than the exposure or intervention of interest?** | **4. Was there a control group?** | **5. Were there multiple measurements of the outcome both pre and post the intervention/exposure?** | **6. Was follow up complete and if not, were differences between groups in terms of their follow up adequately described and analyzed?** | **7. Were the outcomes of participants included in any comparisons measured in the same way?** | **8. Were outcomes measured in a reliable way?** | **9. Was appropriate statistical analysis used?** |  |  |
| --- | --- | --- | --- | --- | --- | --- | --- | --- | --- | --- | --- |
| **Pearlstein et al. (2006)** | Yes | Unclear | Unclear | Unclear | Yes | Yes | Yes | Yes | Yes |  |  |
| **Case series studies** | **1.Were there clear criteria for inclusion in the case series?** | **2. Was the condition measured in a standard, reliable way for all participants included in the case series?** | **3. Were valid methods used for identification of the condition for all participants included in the case series?** | **4. Did the case series have consecutive inclusion of participants?** | **5. Did the case series have complete inclusion of participants?** | **6. Was there clear reporting of the demographics of the participants in the study?** | **7 Was there clear reporting of clinical information of the participants?.** | **8. Were the outcomes or follow up results of cases clearly reported?)** | **9. Was there clear reporting of the presenting site(s)/clinic(s) demographic information?** | **10. Was statistical analysis appropriate?** |  |
| **McCauley et al. (2014)** | Yes | Yes | Yes | Unclear | Unclear | No | Yes | Yes | No | Yes |  |

| **Qualitative studies** | **1. Is there congruity between the stated philosophical perspective and the research methodology?** | **2. Is there congruity between the research methodology and the research question or objectives?** | **3. Is there congruity between the research methodology and the methods used to collect data?** | **4. Is there congruity between the research methodology and the representation and analysis of data?** | **5. Is there congruity between the research methodology and the interpretation of results?** | **6. Is there a statement locating the researcher culturally or theoretically?** | **7. Is the influence of the researcher on the research, and vice- versa, addressed?** | **8. Are participants, and their voices, adequately represented?** | **9. Is the research ethical according to current criteria or, for recent studies, and is there evidence of ethical approval by an appropriate body?** | **10. Do the conclusions drawn in the research report flow from the analysis, or interpretation, of the data?** |  |
| --- | --- | --- | --- | --- | --- | --- | --- | --- | --- | --- | --- |
| **Patel et al. (2005)** | Unclear | Unclear | Unclear | Unclear | Unclear | No | No | Yes | No | Yes |  |
| **Dolman et al. (2006)** | Unclear | Yes | Yes | Yes | Yes | Unclear | Yes | Yes | Yes | Yes |  |
